# DISCOVERING BIOENERGETIC AND FITNESS PROFILES OF COMBINED PERCEIVED AND PERFORMANCE FATIGABILITY

**DOI:** 10.1093/geroni/igae098.0239

**Published:** 2024-12-31

**Authors:** Emma Gay, Caterina Rosano, Paul Coen, Reagan Garcia, Bret Goodpaster, David Marcinek, Anne Newman, Nancy Glynn

**Affiliations:** University of Pittsburgh School of Public Health, Pittsburgh, Pennsylvania, United States; University of Pittsburgh, Pittsburgh, Pennsylvania, United States; AdventHealth, Orlando, Florida, United States; University of Pittsburgh School of Public Health, Pittsburgh, Pennsylvania, United States; Advent Health, Orlando, Florida, United States; University of Washington, Seattle, Washington, United States; University of Pittsburgh, University of Pittsburgh, Pennsylvania, United States; University of Pittsburgh School of Public Health, Pittsburgh, Pennsylvania, United States

## Abstract

Demographic characteristics, skeletal muscle energetics, cardiorespiratory fitness (VO2peak), walking energetics, physical activity, and mobility are all independently associated with perceived and performance fatigability. Perceived and performance fatigability are distinct measurements that are complimentary to each other, and together could provide novel insight into potential targets for healthy aging interventions. We hypothesized that individuals with lower scores on both fatigability measurements (i.e., lower perceived/lower performance) will exhibit better cellular energetics and physical performance profiles compare to individuals with higher scores on both fatigability measurements (i.e., higher perceived/higher performance). Using a sample from SOMMA (n=799), we classified individuals using established severity cutpoints for the perceived-based Pittsburgh Fatigability Scale (PFS, range 0-50, lower<15, higher≥15) and the performance-based Pittsburgh Performance Fatigability Index (PPFI, 0-100%, men: lower<5.4%, higher≥5.4%, women: lower<3.5%, higher≥3.5%). Skeletal muscle energetics were measured in vivo via magnetic resonance spectroscopy and ex vivo (biopsies) via high-resolution respirometry. Daily activity count was measured with wrist-worn accelerometry. We assessed VO2peak during a maximal treadmill cardiopulmonary test and walking energetics (cost-capacity ratio [VO2 from 0.67 m/s 5-min walk/VO2peak*100]). Compared to those with higher PFS/higher PPFI (n=73, 9.1%), individuals classified as lower PFS/lower PPFI (n=331, 41.4%) were 2 years younger, 33% more likely a man, 16% more activity, had 18.5% (0.19 m/s) faster 4m gait speed, 15-25% higher muscle energetics, 26% (5.2 mL/kg/min) higher VO2peak, and 11% lower cost-capacity ratio, all p<0.05. Combining fatigability status revealed striking differences between lower PFS/lower PPFI vs higher PFS/higher PPFI, providing insight into targets (e.g., physical activity, bioenergetics, fitness) for intervention.

